# CD4^+^ T cell heterogeneity in gestational age and preeclampsia using single-cell RNA sequencing

**DOI:** 10.3389/fimmu.2024.1401738

**Published:** 2024-05-07

**Authors:** Sayaka Tsuda, Shigeyuki Shichino, Tamara Tilburgs, Tomoko Shima, Keiko Morita, Akemi Yamaki-Ushijima, Krishna Roskin, Michio Tomura, Azusa Sameshima, Shigeru Saito, Akitoshi Nakashima

**Affiliations:** ^1^ Department of Obstetrics and Gynecology, University of Toyama, Toyama, Japan; ^2^ Division of Immunobiology, Center for Inflammation and Tolerance, Cincinnati Children’s Hospital, Cincinnati, OH, United States; ^3^ Division of Molecular Regulation of Inflammatory and Immune Diseases, Research Institute of Biomedical Sciences, Tokyo University of Science, Chiba, Japan; ^4^ Divisions of Biomedical Informatics & Immunobiology, Cincinnati Children’s Hospital, Cincinnati, OH, United States; ^5^ Laboratory of Immunology, Faculty of Pharmacy, Osaka Ohtani University, Osaka, Japan; ^6^ Ladies’ Clinic We! Toyama, Toyama, Japan; ^7^ University of Toyama, Toyama, Japan

**Keywords:** CD4 + T cell, FOXP3, immune tolerance, preeclampsia, pregnancy, regulatory T cell, single-cell RNA-sequencing, T cell receptor

## Abstract

A balance between pro-inflammatory decidual CD4^+^ T cells and *FOXP3^+^* regulatory T cells (*FOXP3^+^* Tregs) is important for maintaining fetomaternal tolerance. Using single-cell RNA-sequencing and T cell receptor repertoire analysis, we determined that diversity and clonality of decidual CD4^+^ T cell subsets depend on gestational age. Th1/Th2 intermediate and Th1 subsets of CD4^+^ T cells were clonally expanded in both early and late gestation, whereas *FOXP3^+^* Tregs were clonally expanded in late gestation. Th1/Th2 intermediate and *FOXP3^+^* Treg subsets showed altered gene expression in preeclampsia (PE) compared to healthy late gestation. The Th1/Th2 intermediate subset exhibited elevated levels of cytotoxicity-related gene expression in PE. Moreover, increased Treg exhaustion was observed in the PE group, and *FOXP3^+^* Treg subcluster analysis revealed that the effector Treg like subset drove the Treg exhaustion signatures in PE. The Th1/Th2 intermediate and effector Treg like subsets are possible inflammation-driving subsets in PE.

## 1 Introduction

Extravillous trophoblasts, which express allogeneic fetal antigens, come into direct contact with maternal immune cells but are not rejected because of functional alterations in maternal immune cells. While CD8^+^ cytotoxic T cells (CTL) and CD4^+^ T cells, including Th17, Th1, and Th2, are proinflammatory and contribute to immune recognition of fetal allo-antigens, *FOXP3^+^* regulatory T cells (*FOXP3^+^* Tregs) provide immune tolerance. A balance between proinflammatory and tolerogenic immune responses is necessary for maintenance of a pregnancy. *FOXP3^+^* Tregs are a minor population among all decidual immune cells; however, they are crucial for inducing fetal antigen-specific tolerance in allogeneic pregnancies in mice (1–5) and humans (6, 7).

Preeclampsia (PE) is a pregnancy complication characterized by maternal hypertension, proteinuria, and fetal placental circulation failure that is diagnosed after 20 weeks of gestation (8). Because poor placentation is the basis of PE, especially in early onset PE (diagnosed before 34 weeks), immune maladaptation has been proposed to underlie the development of PE (9, 10), and this relationship is supported by many reports. Epidemiologically, the risk of developing PE is higher in cases with inadequate exposure to paternal antigens (first partner pregnancy, long interpregnancy interval, oocyte donation, etc.), possibly due to inadequate induction of paternal antigen-specific immune tolerance (11–15). In addition, quantitative and functional impairment of Tregs has been reported during PE (6, 16–20). *FOXP3^+^* Tregs are a heterogeneous population with multiple subsets. A previous study identified functionally distinct subtypes of *FOXP3^+^* Tregs in human peripheral blood, including FOXP3^low^CD45RA^+^ naïve Tregs, FOXP3^high^CD45RA^-^ effector Tregs, and FOXP3^low^CD45RA^-^ non-suppressive T cells (21). Of these, effector Tregs have the strongest suppressive ability, a memory phenotype, and high expression of ICOS, TIGIT, HLA-DR, ENTPD1, and CTLA-4 (21–23). In contrast, FOXP3^low^CD45RA^-^ non-suppressive T cells have no suppressive ability and low expression of ICOS and TIGIT and are regarded as activated FOXP3^+^ effector CD4^+^ T cells (non-Tregs) (21–23). Decreased frequencies of effector Tregs have been found in the decidua after miscarriages with normal karyotypes (24, 25). Reduced clonal expansion of effector Tregs in the human decidua after PE has also been reported (25). Activation of proinflammatory T cells, including CD4^+^ Th17 cells (26–29), and inadequate suppression of CD8^+^ T cells by PD-1 (30) have also been reported in PE.

Although current single-cell RNA sequencing (scRNA-seq)-based and mass-cytometry-based studies have revealed landscapes of highly heterogeneous maternal immune cells and their populational or phenotypic changes according to gestational age and late onset PE (31–37), the characteristics of decidual Tregs and CD4^+^ T cell subsets and their clonal expansion are not fully described because of their relative rarity.

Therefore, we conducted scRNA-seq and single-cell T cell receptor (TCR) sequencing of decidual CD4^+^T cells during healthy early gestation, healthy late gestation, and PE-associated gestation. We found that decidual CD4^+^ T cells and Treg subsets show differences in gene expression and clonality depending on gestational age and the presence or absence of PE. Most interestingly, we observed extensive clonal expansion of *FOXP3^+^* effector Tregs during late and PE-associated gestation, as well as increased signatures of Treg exhaustion in the *FOXP3^+^* effector Treg like subset in PE.

## 2 Materials and methods

### 2.1 Human subjects

This study was approved by the University of Toyama Institutional Review Board (ID: R2016144 and R20161442). Informed consent was obtained from all participants. Four patients who underwent artificial abortion in the first trimester were enrolled at the Ladies’ Clinic We! Toyama. Three healthy pregnant women and three patients with PE were enrolled at the University of Toyama. One healthy pregnant woman provided peripheral blood and decidua. All abortions were performed by manual vacuum aspiration. All pregnancies in healthy women and preeclamptic patients were delivered via Caesarean section. PE was defined as gestational hypertension accompanied by proteinuria, maternal organ dysfunction, or uteroplacental dysfunction, at or after 20 weeks of gestation (8). Detailed characteristics of the participants are provided in **Supplementary Table 1**.

### 2.2 Cell isolation

First-trimester decidual tissues were isolated from the uterine content obtained by manual vacuum aspiration. The decidua basalis from healthy late gestation and PE deliveries was macroscopically identified and dissected from the maternal surface of the delivered placenta. The samples were processed within 24 h of collection. Decidua was rinsed with phosphate buffered saline (PBS) until the blood was removed, minced with a pair of scissors to produce 1–2 mm pieces, and filtered through 41-μm nylon net filter. The filtered cell suspension was layered with Ficoll Hypaque and centrifuged at 1500 rpm for 30 min with the brakes off. The immune cells at the gradient interface were collected, washed with PBS, and cryopreserved. Peripheral blood mononuclear cells (PBMC), similar to decidual immune cells, were isolated using density gradient centrifugation.

### 2.3 cDNA library construction using the BD Rhapsody system and sequencing

The cryopreserved mononuclear cells were thawed and incubated with the following antibodies for 20 min: PE anti-CD3, APC-Cy7 anti-CD4, APC anti-CD14, PE-Cy7 anti-CD25, FITC anti-CD37, and BD human Sample Tags (**Supplementary Table 2**). CD3^+^CD4^+^CD14^-^CD37^-^cells were sorted into CD4^+^ T cells using a FACSAria II flow cytometer (BD Bioscience, Franklin Lakes, NJ, USA). scTCR/Targeted RNA-seq libraries of CD4^+^ T cells were constructed using a BD Rhapsody system with BD Rhapsody Targeted mRNA and AbSeq Reagent kit (BD Bioscience, Franklin Lakes, NJ, USA), and a BD Rhapsody T Cell Expression Panel Hs (BD Bioscience, Franklin Lakes, NJ, USA) with human *IL-10* and *NRP1* primer supplements (BD Bioscience, Franklin Lakes, NJ, USA) according to the manufacturer’s instructions, except that cDNA denaturation was performed using 0.1M NaOH for 5 min at room temperature. Sequencing was performed using Illumina NovaSeq 6000 and NovaSeq S4 flow cells (200 cycles kit, read 1 67 bp, read 2 155 bp) (Illumina, San Diego, CA, USA) by ImmunoGeneTeqs, Inc. (Chiba, Japan). Details of the key resources are provided in **Supplementary Table 2**.

### 2.4 Fastq data preprocessing and generation of the single-cell gene-expression matrix

Fastq data preprocessing was conducted using ImmunoGeneTeqs, Inc. (Chiba, Japan), as follows. After adapter trimming and quality filtering, the base composition of the sequencing data was analyzed using FastQC-v0.11.9. Pair-ended FASTQ files (R1: cell barcode reads; R2: RNA reads) were processed, and the filtered cell barcode reads were annotated using the Python script provided by BD, with minor modifications for compatibility with Python3.8. The associated cDNA reads were mapped to reference RNA (build GRCh38 release-101). Then, the cell barcode information of each read was added to the bowtie2-mapped BAM files, and the read counts of each gene in each cell barcode were determined using mawk. The cells with a total read count above the inflection point were considered valid. To assign each tag to a cell barcode, the read counts of each tag in each valid cell barcode, defined by the cDNA matrix, were extracted from the tag/cell barcode expression matrix. Unassigned cell barcodes were labeled as “not-detected” cells. Then, the sum of the total read counts of each tag was normalized to 10 M reads and the log2 fold change between the first most counted tags and second most counted tags within each cell barcode. Doublet cells (double-positive cells of any pair of Tags (identified by the flowDensity package)) and log2 fold change between the first and second most counted tags < 0.2415 were determined. Finally, the remaining cell barcodes were assigned to the first most-counted tag. The tag expression in each cell barcode was log2(x+1)-transformed and z-scaled by each cell barcode.

TCR data matrices containing VDJ read counts, nucleotide sequences, amino acid sequences of CDR3, and gene usage were provided by ImmunoGeneTeqs Inc. (Chiba, Japan). Cells with paired alpha/beta chains were used for TCR analysis.

### 2.5 Single-cell data analysis

Distribution-based error correction (DBEC), which included the BD Rhapsody targeted scRNA-seq workflow to reduce background read counts of each gene that were possibly derived from RNA diffusion during the cell lysis step in the BD Rhapsody cartridge, and reverse transcription were conducted by ImmunoGeneTeqs, Inc. (Chiba, Japan).

We created a Seurat object, in which two batches were combined. We calculated metrics (percent mito, percent ribo) and filtered out mitochondrial gene-high cells (> 0.25), doublets and non-hashtag-assigned cells. Because BD Rhapsody-targeted data are non-UMI data (raw count data), we performed global normalization to 1 M tags and scaling using the Seurat ScaleData function by regressing out the library size (total raw read count of each cell) as a confounding factor. We split the combined Seurat objects into a list, with each batch considered an element. We identified variable features individually for each using the FindVariableFeatures function with default parameters. Next, we identified the anchors for each batch and integrated them using the FindIntegrationAnchors function with 50 dimensions. These anchors were then passed to the integrated data function. We used this integrated matrix for the downstream analysis. Dimension reduction was performed using the RunPCA function on an integrated object with the first 50 PCA dimensions. Clusters were visualized using FindNeighbors, FindClusters, and RunUMAP functions with the top 15 dimensions (resolution set to 0.8). Differential markers between the clusters were detected using the FindAllMarker function. Cell types were determined according to previously reported scRNA markers for T cells from peripheral blood, decidua, and cancers (33, 34, 38–40). Module scores of Treg exhaustion-related genes (*TNFRSF18, TNFRSF4, PDCD1, IFNG, LAG3, CCR5, CXCR3, CCR6, HAVCR2, CXCR6, NKG7, SLAMF1, KLRB1, CD2*, and *CXCR1*) were calculated as previously reported (39, 41) using the AddModuleScore function. Differential expression analysis was conducted using the FindMarkers function with default settings. Software and algorithms are listed in **Supplementary Table 2**. Genes with log fold change > |0.5| and adjusted p-values ≤ 0.05 were regarded statistically significant.

### 2.6 Enriched Gene Ontology analysis

We uploaded the list of differentially expressed genes (DEGs) identified using the FindMarkers function in Seurat to the Matascape (https://metascape.org/gp/index.html#/main/step1) main page and chose “Express Analysis.”

## 3 Results

### 3.1 Decidual CD4^+^ T cells are heterogenous

To assess decidual CD4^+^ T cell diversity, we conducted a single-cell transcriptome analysis of CD4^+^ T cells isolated from the decidua basalis of healthy early gestation (6–7 weeks, n = 4), healthy late gestation (37–40 weeks, n = 3), and gestation with early onset PE diagnosed before 34 weeks who delivered between 34 and 37 weeks of gestation (n = 3) (**Supplementary Table 1**). Women with autoimmune diseases were excluded from this study. CD4^+^ T cells from PBMC from one healthy late gestation patient were also included. CD4^+^ T cells were sorted and subjected to a BD Rhapsody Single-Cell Analysis (**Supplementary Figure 1A**). Dead cells and doublets were removed from the scRNA-seq data. Dimension reduction and clustering were performed on 24,002 cells that passed quality control (QC) tests.

**Figure 1:**
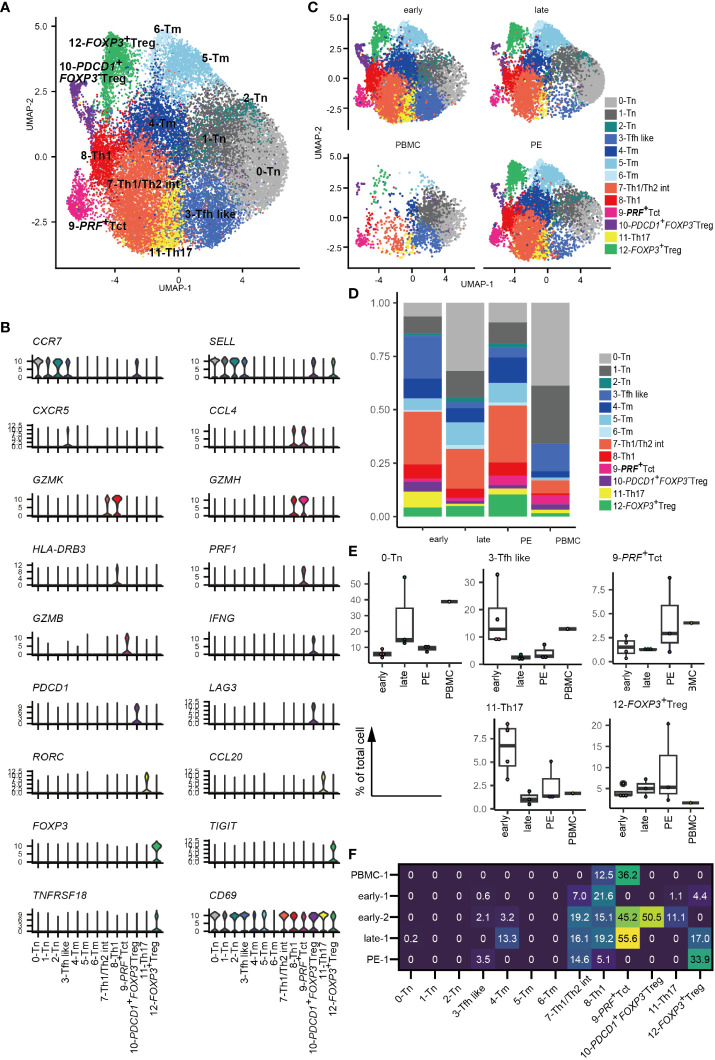
Landscape of decidual CD4^+^ T cells. **(A)** UMAP plot of 24,002 cells from 11 samples showing CD4^+^ T cell subsets. Thirteen distinct clusters were annotated using gene expression markers. **(B)** Violin plots represent the normalized expression levels (y-axis) of key cluster-defining genes in CD4^+^ T cells. **(C)** UMAP plots of CD4^+^ T cell subsets based on sample origin: healthy early gestation decidua (n = 4, 8472 cells) (upper left), healthy late gestation decidua (n = 3, 6572 cells) (upper right), healthy late gestation PBMC (n = 1, 2628 cells) (lower left), and PE decidua delivered at term (n = 3, 6330 cells) (lower right). **(D)** Stacked bar graph comparing the distribution of CD4^+^ T-cell clusters based on sample origin. **(E)** Floating box plots showing the abundance of cells in 0-Tn, 3-Tfh like, 9-*PRF^+^* Tct, 11-Th17, and 12-*FOXP3^+^* Treg clusters. **(F)** Heatmap showing percentages of expanded clones (size = 2 or more cells) in each cluster. The columns indicate CD4^+^ T cell clusters. Rows indicate sample origins.

Thirteen CD4^+^ T cell clusters were identified based on cluster marker feature genes and previously reported scRNA-seq data for CD4^+^ T cells (33, 38, 39) (**Figures 1A, B**, **Supplementary Figure 1B**, **Supplementary Table 3**). CD4^+^ T cells included i) three clusters of *CCR7*- and *SELL*-expressing naïve (0–2-Tn), ii) one *CXCR5^+^* follicular T cell like cluster (3-Tfh like), iii) three clusters of memory CD4^+^ T cells (4–6-Tm), iv) one cluster of Th1/Th2 intermediate cells expressing *GZMK* and *TBX21*, which suggests Th1 type, as well as *GATA3*, which suggests Th2 type (7-Th1/Th2 int); v) one cluster of Th1-type cells expressing high levels of *GZMK, HLA-DR*, and *TBX21* (8-Th1), vi) one cluster of activated CD4^+^ T cells with high *PRF1* expressing activated T cells (9-*PRF^+^* Tct); vii) one cluster of *FOXP3^-^* CD4^+^ T cells expressing *PDCD1, LAG3*, and *IFNG* (10-*PDCD1^+^FOXP3^-^* Treg) with a gene expression signature similar to that of FOXP3^-^PD-1^+^ Tregs reported previously in decidua (42), viii) one cluster of Th17-type cells with high *RORC* expression (11-Th17); and viii) one cluster of *FOXP3^+^* Treg also expressing *TIGIT*, *IL2RA, TNFRSF18* (also known as *GITR*) and *ENTPD1* (also known as *CD39*) (12-*FOXP3^+^* Treg) in concordance with prior studies (42) (**Figures 1A, B**, **Supplementary Figure 1B**). *CD69* was expressed across all clusters of CD4^+^ T cells, with the exception of 6-Tm cells (**Figure 1B**), suggesting their residence in tissues as described previously (33, 35, 43). Thus, decidual CD4^+^ T cell populations were found to be heterogeneous and include distinct types of activated memory, helper, cytotoxic, and regulatory T cells.

### 3.2 Decidual CD4^+^ T cell cluster frequencies differ among healthy early, healthy late and PE pregnancies

Next, CD4^+^ T cells were separated into early and late gestation and PE and PBMC groups (**Figures 1C-E**). Decidual CD4^+^ T cells contained more abundant populations of memory, 7-Th1/Th2 int, 8-Th1, 9-*PRF^+^* Tct, 10-*PDCD1^+^FOXP3^-^* Treg, 11-Th17, and 12-*FOXP3^+^* Treg than PBMC (**Figures 1C–E**). The frequency of CD4^+^ T cell subsets did not differ significantly among healthy early, healthy late, and PE groups because of their small sample sizes and inter-patient heterogeneity. However, an increased tendency of 3-Tfh like and 11-Th17 cells was observed in early gestation, whereas late gestation had a modest increase in tendency toward enrichment with the 0-Tn group (**Figure 1E**). Interestingly, decidual CD4^+^ T cells from PE samples showed an increased tendency toward 9-*PRF^+^* Tct cells compared to cells found in late gestation (**Figure 1E**). The frequency of decidual 12-*FOXP3^+^* Treg varied in PE (**Figure 1E**), as a recent meta-analysis also describes (44). Thus, changes in CD4^+^ T cell cluster frequencies are related to gestational age and the presence or absence of PE.

### 3.3 Clonally expanded CD4^+^ T cells are found in Th1/Th2 int, Th1, *PRF^+^* Tct, and *FOXP3^+^* Treg clusters

**Figure 2:**
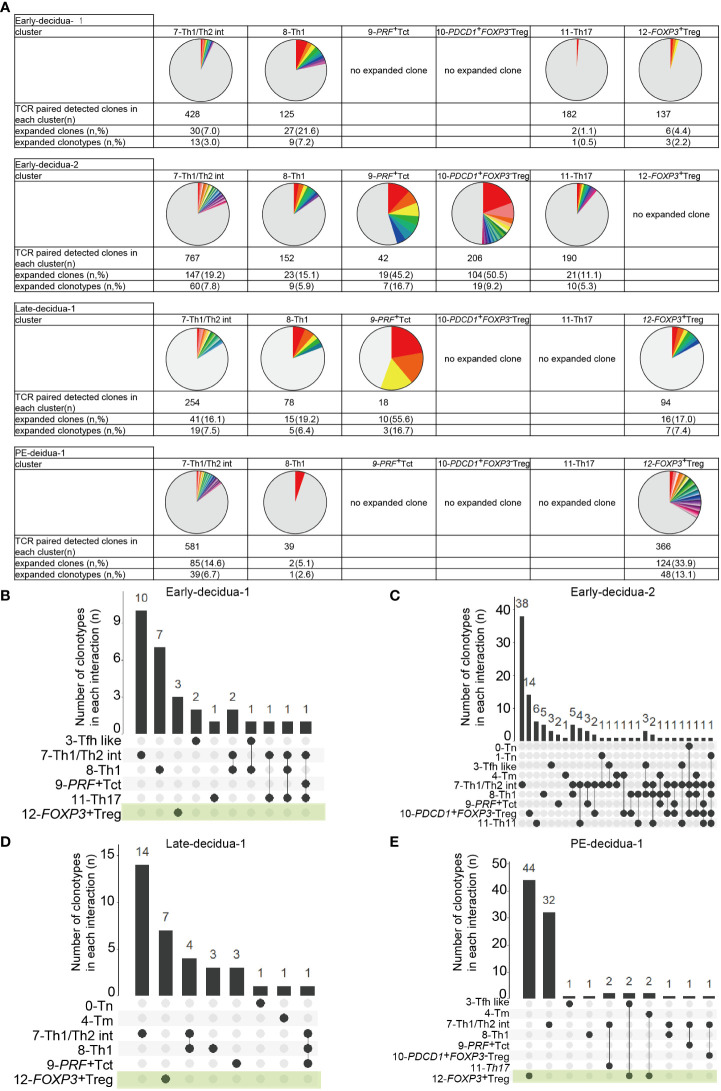
Distributions of expanded clonotypes in each CD4+ T cell cluster. **(A)** Pie charts show the ratio of expanded clones (n ≥ 2 clones) in each cluster. Gray parts represent unique clonotypes (n = 1 clones). Colored parts represent expanded clonotypes. The same color does not mean the same clonotype. **(B–E)** UpSetR graphs showing inter-cluster overlap of clonotypes that have clone sizes of 2 or more for each subject. Histogram bar (top) shows number of clonotypes shared between individual clusters (bottom left). Each dot (bottom) represents a cluster in which clonotypes exist. Connected lines represent clusters that share clonotypes.

Next, we conducted a TCR analysis to assess clonal expansion within each cluster. Gene expression and TCR repertoire data were combined from 14,020 cells of five subjects (two early gestational decidua, one late gestational decidua, one PE decidua, and one PBMC sample). In total, 13,538 (96.6%) TCR alpha/beta-paired cells were detected. Clonal expansion was primarily observed in the 7-Th1/Th2 int, 8-Th1, 9-*PRF^+^* Tct, and 12-*FOXP3^+^* Treg clusters (**Figures 1F**, **2A**). 10-*PDCD1^+^FOXP3^-^* Treg was clonally expanded in one early gestation subject (early-decidua-2) (**Figures 1F**, **2A**). Clonal expansions in 7-Th1/Th2 int and 8-Th1 were observed in early, late gestation and in PE (**Figures 1F**, **2A**). Clonal expansion of 12-*FOXP3^+^* Treg was observed in late gestation and PE, but not in early gestation (**Figures 1F**, **2A**), which is consistent with our previous report (25). Inter-cluster overlap of expanded clonotypes (clone size = 2 or more) showed that the majority of expanded clones belonged to one cluster, whereas a few had multiple phenotypes (**Figures 2B-E**), confirming that TCR usage partly shapes the CD4^+^ T cell phenotype, as previously described (38, 45–47). Fewer shared clones with Tregs and CD4^+^ Th subsets have been reported in term decidua (48). We confirmed that clonally expanded cells in the 12-*FOXP3^+^* Treg cluster shared fewer overlapping clones with other clusters (**Figures 2B–E**). In contrast, some clones overlapped among 7-Th1/Th2 int, 8-Th1, 9-*PRF^+^* Tct, 11-Th17, and memory T cell subsets (**Figures 2B–E**).

Collectively, these results indicated that decidual CD4^+^ T cells are phenotypically and clonally heterogeneous. Naïve, *CXCR5^+^* Tfh like, memory, Th1, *PRF^+^* Tct, Th17, *FOXP3^-^PD-1^+^* Tregs, and *FOXP3^+^* Treg clusters observed here were consistent with previously reported clusters confirmed by gene expression and flowcytometry (33, 38, 39, 42); however, 7-Th1/Th2 was not identified in previous reports. Despite inter-subject heterogeneity, clonal expansion was predominantly observed in 7-Th1/Th2 int, 8-Th1, 9-*PRF^+^* Tct, and 12-*FOXP3^+^* Treg, but not in naïve clusters in the donors analyzed. The 8-Th1, 9-*PRF^+^* Tct, 11-Th17, and 7-Th1/Th2 clusters shared some clones, suggesting that they also share some differentiation processes. However, the major expanded clones were unique to each cluster (**Figures 2B–E**), supporting the validity of the presence of Th1/Th2 clusters in the decidua. The distinct TCR repertoire between 12-*FOXP3^+^* Treg and CD4^+^ T cell subsets suggests that decidual *FOXP3^+^* Tregs have clonal origins different from those of decidual CD4^+^ Th subsets.

### 3.4 Decidual CD4^+^ effector, memory, and Tregs have decreased signatures of lymphocyte activation in late gestation compared to early gestation

**Figure 3:**
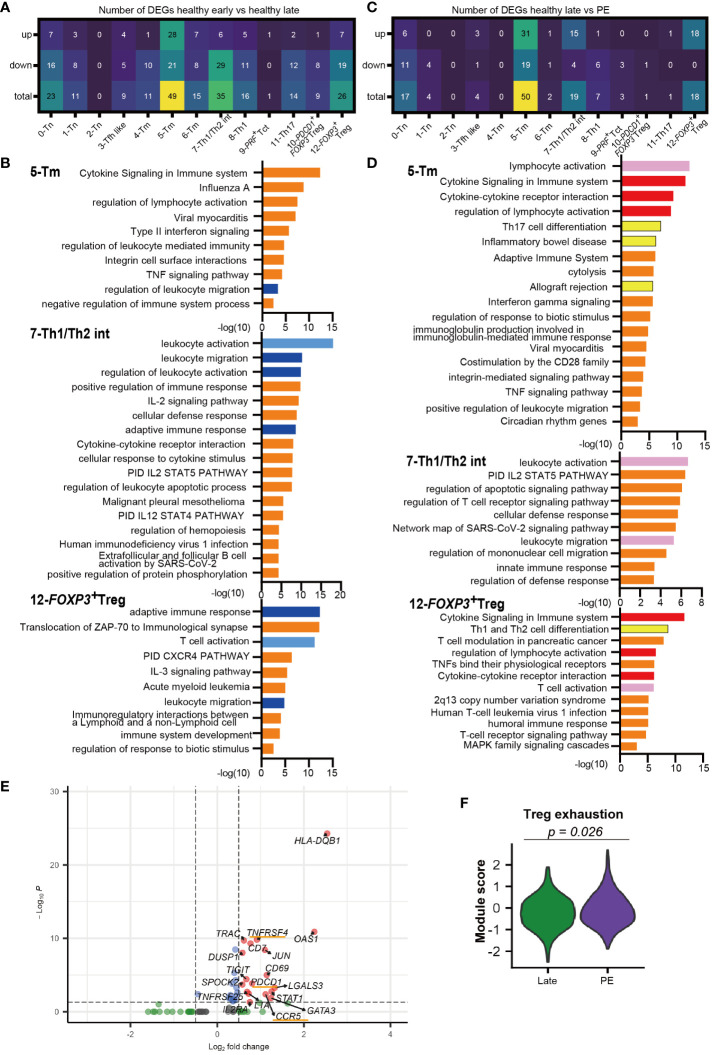
Different transcriptomic signatures between healthy early vs. healthy late and healthy late vs. PE decidua. **(A)** Number of DEGs that are upregulated or downregulated in healthy late gestation compared to healthy early gestation decidua. DEG: adjusted p-value < 0.05, |log2 fold change| > 0.5. **(B)** GOs of downregulated genes in 5-Tm, 7-Th1/Th2 int, and 12-*FOXP3^+^* Treg clusters identified by Metascape in healthy late decidua compared to healthy early decidua. Shared GOs between clusters and similar GOs are marked with blue and sky blue, respectively. **(C)** Number of DEGs that are upregulated or downregulated in PE compared to healthy late gestation decidua. DEG: adjusted p-value < 0.05, |log2 fold change| > 0.5. **(D)** GOs of upregulated genes in 5-Tm, 7-Th1/Th2 int, and 12-*FOXP3^+^* Treg clusters identified by Metascape in PE compared to healthy late decidua. Shared GOs between clusters, similar GOs, and unique key GOs are marked with red, pink, and yellow, respectively. **(E)** Volcano plot representing DEGs in the 12-*FOXP3^+^* Treg cluster shared between healthy late gestation and PE. **(F)** Violon plot showing the module score of Treg exhaustion in the 12-*FOXP3^+^* Treg cluster from term and PE (Mann–Whitney U test).

Distinct decidual T-cell frequencies and transcriptomic signatures in early compared to late gestation have been reported previously (33, 35). However, the gestational age-dependent transcriptomic differences in CD4^+^ T cells are not well understood. An analysis of DEGs between early and late gestation was performed for each CD4^+^ T cell cluster. Enriched GOs from the DEGs were analyzed using Metascape (49). Most CD4^+^ T cell clusters had more downregulated genes in late than early gestation, and DEGs were particularly prevalent in the 5-Tm, 7-Th1/Th2 int, and 12-*FOXP3^+^* Treg clusters (**Figure 3A**, **Supplementary Figures 2A–C**). Focusing on the GOs of downregulated genes in healthy late gestation compared to early gestation in these three clusters, similar GOs were shared across the clusters (**Figure 3B**). In particular, GOs related to migration, such as leukocyte migration and regulation of leukocyte migration, were shared among the 5-Tm, 7-Th1/Th2 int, and 12-*FOXP3^+^* Treg clusters (**Figure 3B**). In addition, GOs related to activation, such as leukocyte activation, T cell activation, and adaptive immune response, were downregulated in 7-Th1/Th2 int and 12-*FOXP3^+^* Treg in late gestation (**Figure 3B**). Genes involved in leukocyte activation, such as *NKG7* and *PRF1*, and those involved in adaptive immune responses, such as *TRAC* and *CD3G*, were downregulated in 7-Th1/Th2 int in late gestation (**Figure 3B**, **Supplementary Figure 2B**, **Supplementary Table 4**). In 12-*FOXP3^+^* Treg, *LGALS1* and *CTLA4*, which are related to Treg function, were upregulated in late gestation, whereas more genes related to T cell activation and adaptive immune response, such as HLA-class II molecules, were downregulated in late gestation (**Figure 3B**, **Supplementary Figure 2C**, **Supplementary Table 4**).

In summary, this study revealed significant differences in gene expression of CD4^+^ T cells between healthy early and late decidua. Specifically, 5-Tm, 7-Th1/Th2 int, and 12-*FOXP3^+^* Treg in healthy late gestation had substantially downregulated gene sets associated with leukocyte activation and migration compared to those in early gestation. Interestingly, 12-*FOXP3^+^* Treg showed downregulation of genes involved in cell activation and immune responses; however, they showed upregulation of genes related to Treg function in late gestation.

### 3.5 Decidual CD4^+^ effector, memory, and Tregs have increased proinflammatory signatures in PE-associated compared to healthy late gestation

Next, we compared DEGs between the healthy late gestation and PE groups to determine transcriptomic changes in T cell clusters. DEG analysis showed that genes upregulated in PE were mainly observed in clusters 5-Tm, 7-Th1/Th2 int, and 12-*FOXP3^+^* Treg, which were consistent with the clusters that had the most DEGs in the early and late comparisons (**Figures 3C, E**, **Supplementary Figures 2D, E**). Next, GO analysis of upregulated genes with PE in the 5-Tm, 7-Th1/Th2 int, and 12-*FOXP3^+^* Treg clusters revealed that activation-related GOs (lymphocyte activation, leukocyte activation, and T cell activation) were shared among the three clusters (**Figure 3D**). The cytokine signaling and regulation of lymphocyte activation pathways were upregulated in 5-Tm and 12-*FOXP3^+^* Treg in PE (**Figure 3D**). In 7-Th1/Th2 int, leukocyte activation pathways, including *NKG2* and *PRF1*, were activated in PE (**Figure 3D**, **Supplementary Figure 2E**). In 5-Tm, GOs including Th17 differentiation pathway, inflammatory bowel disease, and allograft rejection, represented by *IL12RB1*, *IRF4*, *LAT*, and *IL22*, were observed in PE (**Figure 3D**, **Supplementary Figure 2D**, **Supplementary Table 4**). Previous studies have shown excessive Th17 cells in PE (26–29); however, in our study, the Th17 cluster did not show upregulated genes with PE. In addition, neither 5-Tm nor 11-Th17 cells showed clonal expansion in PE (**Figure 1F**), suggesting that a pro-inflammatory environment, and not antigen stimulation, may contribute to increased Th17 signatures in decidual CD4^+^ T cells.

Interestingly, gene sets associated with Th1 and Th2 differentiation, including *GATA3* and *STAT1*, were upregulated in 12-*FOXP3^+^* Treg from the PE group (**Figures 3D, E**). *STAT1* overexpression (39) and *GATA3* overexpression in *FOXP3^+^* Tregs (50, 51) have been reported in autoimmune diseases. Furthermore, *PDCD1*, *CCR5*, and *TNFRSF4* were upregulated only in 12-*FOXP3^+^* Treg in the PE group (**Figure 3E**). Previous reports have shown that these genes are upregulated in exhausted Tregs with reduced suppressive function in autoimmune disease (39, 41). Another report showed that PD-1^high^ exhausted Tregs were significantly increased in the peripheral blood of patients with PE (52). Notably, the module score of Treg exhaustion-related genes (39, 41) was significantly higher in PE than in healthy late gestation (**Figure 3F**).

Taken together, 7-Th1/Th2 int and 5-Tm were activated and showed a proinflammatory signature, whereas 12-*FOXP3^+^* Treg showed an exhausted signature in PE.

### 3.6 *FOXP3^+^* Treg sub-clusters contain Naïve Treg, *FOXP3^+^* effector Treg-like, and *TIGIT* low Treg populations

**Figure 4:**
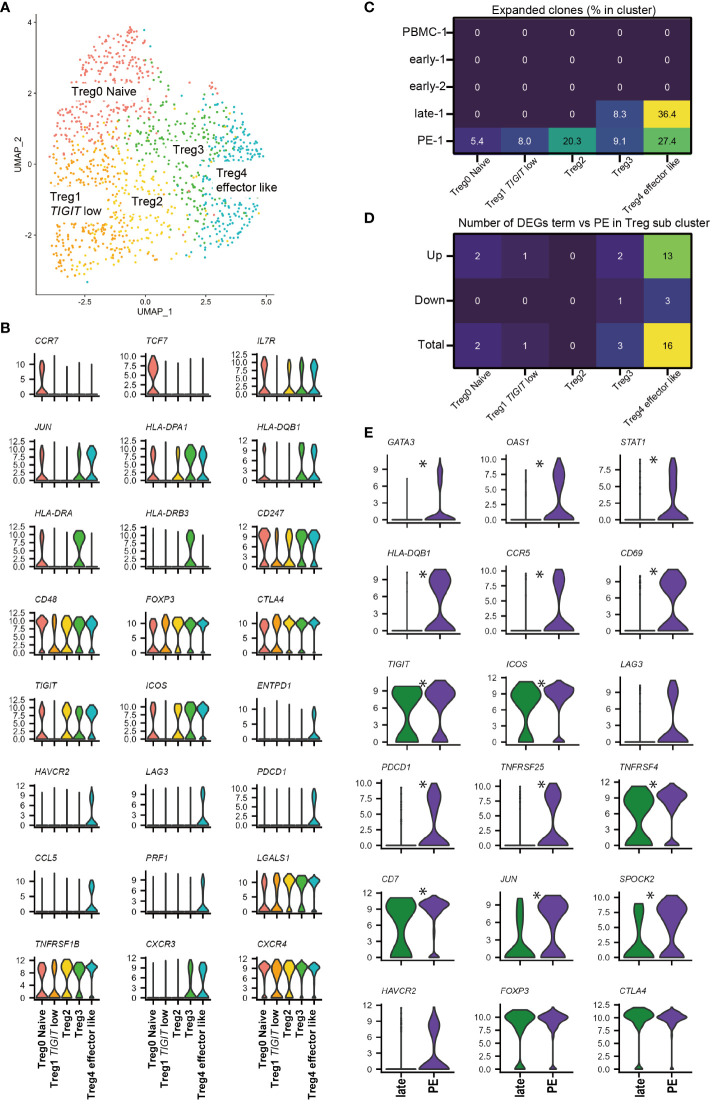
*FOXP3^+^* Treg sub-cluster analysis and alternative Treg functional signature in PE. **(A)** UMAP plot of 1354 cells from 11 samples showing Treg subsets. Five distinct sub-clusters were annotated based on unique gene-expression markers. **(B)** Violin plots representing the normalized expression level (y-axis) of key cluster-defining genes in the *FOXP3^+^* Treg sub-clusters. **(C)** Heatmap showing percentages of expanded clones (size = 2 or more cells) in each cluster. Columns indicate *FOXP3^+^* Treg sub-clusters. Rows indicate sample origins. **(D)** Heatmap showing the number of DEGs that are upregulated or downregulated in PE compared to healthy late gestation decidua in each Treg sub-cluster. DEG: adjusted p-value < 0.05, |log2 fold change| > 0.2. **(E)** Violin plots showing selected genes in Treg4 effector like cluster compared with healthy late and PE. * represents differentially expressed genes (adjusted p-value < 0.05, |log2 fold change| > 0.5) in the whole 12-*FOXP3^+^* Treg cluster or Treg4 effector like cluster subset.

Next, we investigated the *FOXP3^+^* Treg sub-cluster by further analyzing all 1354 cells from 11 samples (**Figure 4A**). Although *CD45RA* was not detected, five *FOXP3^+^* Treg sub-clusters were identified that showed similarity in marker gene expression with previously identified FOXP3^low^CD45RA^+^ naïve Treg, FOXP3^high^CD45RA^-^ effector Treg, and FOXP3^low^CD45RA^-^T cells (21–23, 38–40, 53). *CCR7*, *SELL*, and *TCF7* were highly expressed in Treg0 Naïve cluster, similar to naïve Tregs, as previously described. Treg1 *TIGIT* low cluster had low levels of *TIGIT* and *ICOS*, similar to FOXP3^low^CD45RA^-^ T cells. Treg4 effector like cluster had high levels of *ICOS*, *TIGIT*, *ENTPD1*, and *CTLA-4*, similar to effector Tregs (**Figure 4B**, **Supplementary Figure 3A**). The Treg4 effector like cluster expressed exhaustion-related genes such as *HAVCR2*, *LAG3*, and *PDCD1*; however, they also showed the highest expression of soluble suppressors such as *PRF1* and *LGALS1* (**Figure 4B**, **Supplementary Figure 3A**), supporting Treg4 effector like cluster as an effector and Treg-like properties. The effector Treg like subset, naïve Treg subset, and FOXP3^low^CD45RA^-^ T cell subset in decidua were previously reported by flowcytometry based studies (24, 25, 35). In addition, expanded clones were predominantly observed in Treg4 effector like cluster in the healthy late (36.4%) and PE (27.4%) (**Figure 4C**), consistent with our previous report (25). Treg2 and Treg3 had intermediate characteristics of Treg1 Naive and Treg4 effector like subsets in terms of Treg-specific marker genes; however, Treg3 showed higher expression of HLA-class II molecules than Treg2 (**Figure 4B**, **Supplementary Figure 3A**). Some scRNA-seq-based studies have identified naïve Treg-like subsets, effector Treg-like subsets, and various other undefined subsets (38–40, 53). The functions of these Treg subsets should be defined in future studies. The Treg0 Naïve cluster was abundant in PBMC, whereas Treg2, Treg3, and Treg4 effector like clusters were abundant in the decidua (**Supplementary Figures 3B–G**). Cells in the Treg 4 effector like subset were abundant during late gestation (**Supplementary Figure 3G**). Thus, decidual Tregs are heterogeneous, and changes in cluster frequencies depend on gestational age.

### 3.7 Treg4 effector like subcluster drives alternative Treg functional signature in PE

Next, we investigated whether a specific *FOXP3^+^* Treg cluster drove the Treg exhaustion signature in PE samples (**Figure 3F**) by performing DEG analysis of Tregs in late healthy and PE samples. The number of upregulated genes in PE was highest in the Treg4 effector like subset (**Figure 4D**), and DEG analysis of Treg4 effector like cells showed that Treg exhaustion-related genes (*PDCD1*, *TNFRSF25*, *TNFRSF4*, *CCR5*, *LAG3*, and *HAVCR2*) (39, 41) were more highly expressed in PE than in late healthy pregnancy (**Figure 4E**). Increased expression of *STAT1* and *GATA3* in PE samples was also observed in Treg4 effector like cluster (**Figure 4E**). Based on the results of our TCR clonality and DEG analyses, the Treg4 effector like clusters may reflect antigen stimulation, driving the Treg exhaustion signature in PE.

## 4 Discussion

At the fetomaternal interface, a delicate balance between proinflammatory CD8^+^ CTLs and CD4^+^ T cells, which can respond to fetal antigens, and Tregs, which impart immune tolerance, is required. From the viewpoint of T cell immunity in PE, an imbalance between Th17 and Tregs (26–29), insufficient effector Treg expansion (25), Treg dysfunction (18), and insufficient suppression of CTLs (30) have been reported. In this study, we showed 1) the diversity of decidual CD4^+^ T cell subsets and clonality dependence on gestational age, 2) altered gene expression in Th1/Th2 int, Treg, and memory CD4^+^ T cells in PE, and 3) increased signatures of Treg exhaustion in PE compared to that in healthy late gestation pregnancies.

Decidual CD4^+^ T cells are a heterogeneous population consisting of naïve, memory, effector (Th1/Th2 int, Th1, *PRF^+^* Tct, and Th17), *PDCD1^+^FOXP3^-^* Treg, and *FOXP3^+^* Treg. All the clusters other than Th1/Th2 int were consistent with previously reported characteristics of CD4 T cell subclusters (33, 38, 39, 42). Newly identified Th1/Th2 int cluster expressed Th1 and Th2 signature genes simultaneously. Clonal expansion was mainly observed in Th1/Th2 int, Th1, *PRF^+^* Tct, *PDCD1^+^FOXP3^-^* Treg, and Treg cells, suggesting that these T cell types are involved in antigen-specific responses. The fewer shared clones between Tregs and other CD4^+^ T cell subsets suggest distinct clonal origins for these populations. By contrast, the majority of expanded clonotypes were unique to each Th1/Th2 int, Th1, *PRF^+^* Tct, *PDCD1^+^FOXP3^-^* Treg, and Th17 subsets, some clones were shared between these clusters. This result supports the theory that effector T cell subsets are shaped by TCR sequences, and that each subset is a continuum phenotype (38, 47). We could not validate the Th1/Th2 int cluster by flowcytometry; however, this subset had a distinctive TCR repertoire, supporting that it has a unique phenotype that is different from that of other helper CD4^+^ T cell subsets. *PDCD1^+^FOXP3^-^* Tregs express exhaustion-associated molecules and *IFNG*. This unique signature is similar to that of FOXP3^-^ PD1^HI^ Treg as previously reported (42).

The Th1/Th2 int, *FOXP3^+^* Treg, and Tm subsets had more DEGs than the other CD4^+^ T cell types when healthy early- and late-gestation samples were compared. In the Th1/Th2 int subset, activation- and migration-related genes are downregulated in late gestation samples. Because the Th1/Th2 int subset had clonally expanded populations, it is reasonable to expect that this subset would be suppressed in late gestation. Although *FOXP3^+^* Tregs showed downregulation of genes related to T cell activation and migration, *CTLA-4* and *LGALS1* were upregulated in late gestation samples. In addition, clonal expansion of Tregs appeared late rather than early in gestation, which is consistent with results of our previous study (25). These results suggested that the *FOXP3^+^* Treg subset underwent antigen-responsive functional changes leading up to late gestation.

Interestingly, the Th1/Th2 int subset, which had downregulated activation-related genes in late gestation compared to early gestation, showed elevated expression of T cell activation-related genes in PE, whereas *FOXP3^+^* Tregs overexpressed the Treg exhaustion-related genes *STAT1* and *GATA3* in PE. Altered gene expression in *FOXP3^+^* Tregs from patients with PE was driven by a clonally expanded Treg4 effector like cluster. In peripheral blood from PE patients, PD-1^high^exhausted Treg cells were increased (52). Furthermore, elevation of exhausted Tregs with impaired suppressive abilities have been reported in systemic lupus erythematosus (SLE), inflammatory bowel disease, and malignant tumors (39, 41). In SLE, elevated levels of *STAT1* and other exhaustion related molecules in Tregs are associated with disease severity (39, 54). In addition, a relationship between excessive type 1 interferon (IFN) signaling and Treg exhaustion has been suggested in SLE (39). Our data did not show elevated excessive type 1 IFN signaling pathway in Tregs of PE; however, overexpression of *STAT1* and Treg exhaustion-related genes in PE was consistent with that in SLE. *GATA3*-overexpressing Tregs have been observed in inflamed intestines, which contribute to the accumulation of Tregs in inflamed tissues (50, 51). The DEGs of Tregs in PE overlapped with those in SLE even though samples from patients with autoimmune diseases were excluded in this study. Pregnancies complicated with SLE are at a high risk of developing PE (55–59).Vice versa, a differential DNA accessibility analysis of CD4^+^ T cells from patients with SLE included eclampsia as a disease-related ontology (39), suggesting common underlying CD4^+^ T cell abnormalities in SLE and PE.

A major limitation of this study was its small sample size. In addition, the newly identified subset has not been validated by flow cytometry since there are some molecules with mismatched protein expression and gene expression patterns. A validation cohort study using paired scRNAseq and flowcytometry is desirable. In addition, functional analysis of Tregs in PE will provide further insights into the mechanisms underlying Treg exhaustion.

In summary, we identified clonally expanded Th1/Th2 int and *FOXP3^+^* effector Treg subsets with altered PE gene expression. The Th1/Th2 int subset is activated, whereas the Treg subset is exhausted in PE. Further studies characterizing functionally altered CD4^+^ T-cell subsets and Tregs will accelerate the development of immunological treatments for PE.

## Data availability statement

The datasets presented in this study can be found in online repositories. The names of the repository/repositories and accession number(s) can be found below: The DNA Data Bank of Japan (DDBJ)/PRJDB17285.

## Ethics statement

The studies involving humans were approved by University of Toyama Institutional Review Board. The studies were conducted in accordance with the local legislation and institutional requirements. The participants provided their written informed consent to participate in this study.

## Author contributions

ST: Writing – review & editing, Writing – original draft, Validation, Resources, Methodology, Investigation, Funding acquisition, Formal Analysis, Conceptualization. SSh: Writing – review & editing, Investigation, Formal Analysis. TT: Writing – review & editing. TS: Writing – review & editing, Funding acquisition. KM: Writing – review & editing, Resources, Investigation. AY-U: Writing – review & editing, Investigation. KR: Writing – review & editing. MT: Writing – review & editing. AS: Writing – review & editing, Resources. SSa: Writing – review & editing, Supervision, Methodology, Funding acquisition, Conceptualization. AN: Writing – review & editing, Supervision, Methodology, Funding acquisition.
