# Supplementary material for: CD4+ T cell heterogeneity in gestational age and preeclampsia using single-cell RNA sequencing
Source: Front Immunol. 2024 May 7;15:1401738. doi: 10.3389/fimmu.2024.1401738 (PMC11106458; doi:10.3389/fimmu.2024.1401738)
Supplement: Supplementary file 1 [file Image_1.pdf]

Supplementary Figure 1

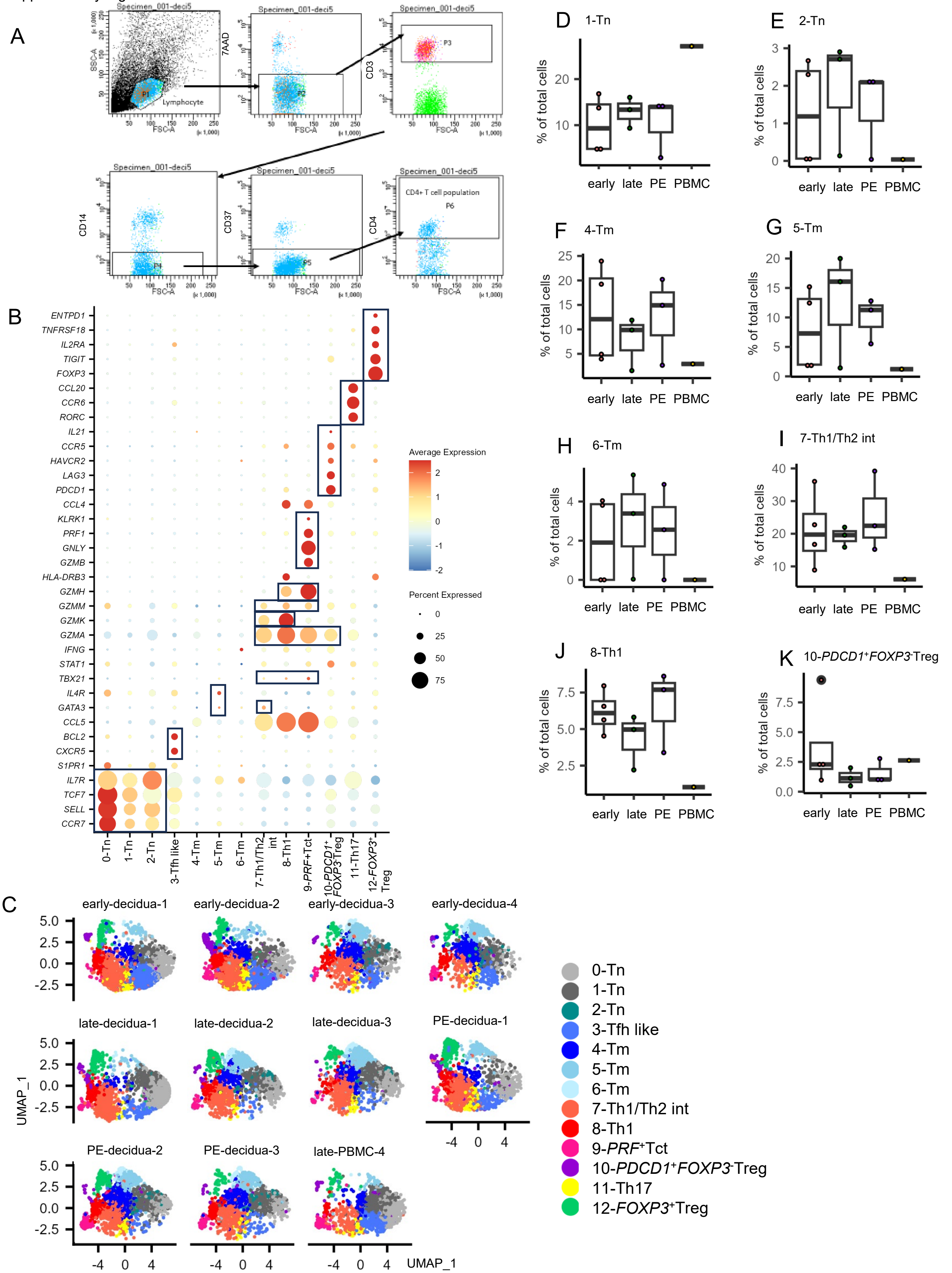

Supplementary Figure 1. Gating strategy for cell sorting and CD4$^+$ T cell subsets.

(A) Gating strategy of sorting CD4$^+$ T cells. (B) Dotplot of key cluster defining genes for CD4$^+$ T cell clusters. Representative cluster defining genes were marked by boxes. (C) UMAP plots of the composition of CD4$^+$ T cells are displayed for each patient origin. (D)-(K) Floating box plots show the abundance of cells in each CD4$^+$ T cell cluster.

Supplementary Figure 2

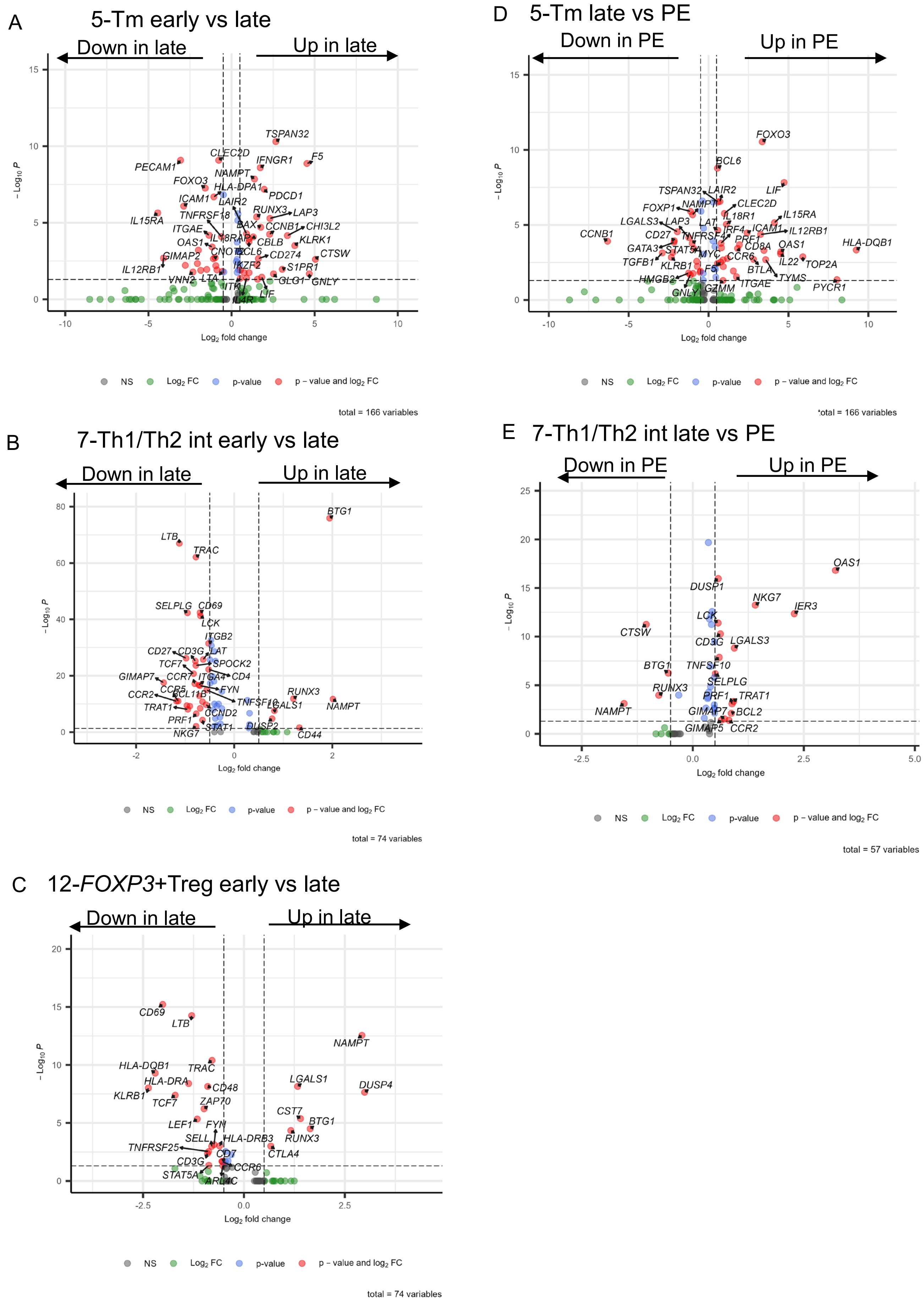

Supplementary Figure 2. Differentially expressed genes between healthy early vs. healthy late and healthy late vs. PE decidua.

(A)-(C) Volcano plots represent DEGs in 5-Tm (A), 7-Th1/Th2 int (B), and 12-*FOXP3*$^{+}$Treg cluster (C) between healthy early gestation and healthy late gestation. (D)-(E) Volcano plots represent DEGs in 5-Tm (D) and 7-Th1/Th2 int (E) between healthy late gestation and PE.

Supplementary Figure 3

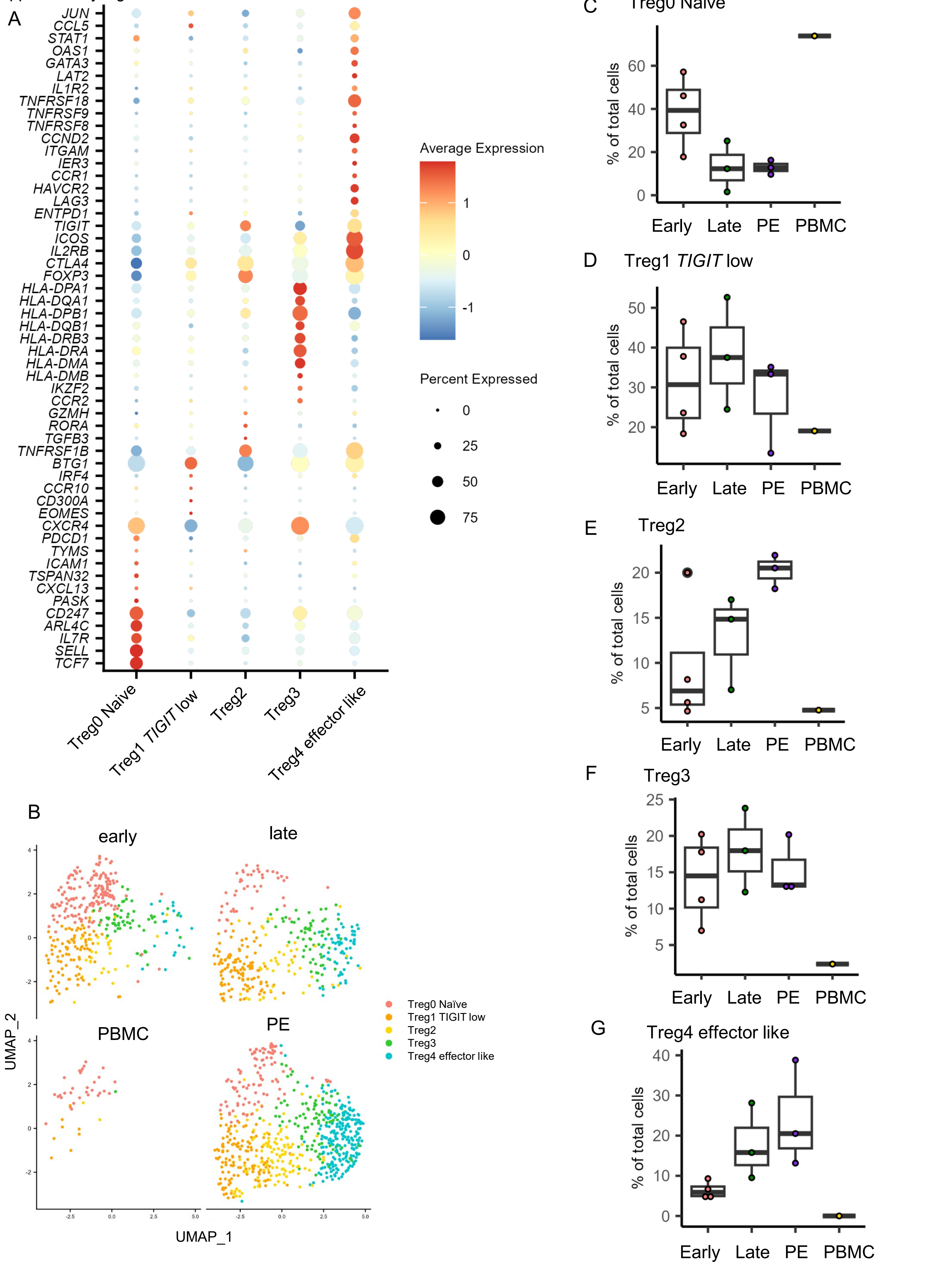

Supplementary Figure 3. Detailed characteristics of *FOXP3*$^{+}$ Treg sub-clusters.

(A) Dotplot of key cluster defining genes for *FOXP3*$^{+}$ Treg sub clusters. (B) UMAP plots of the composition of *FOXP3*$^{+}$ Treg sub clusters are displayed for each sample origin; healthy early gestation decidua (n=4, 364 cells) (upper left), healthy late gestation decidua (n=3, 332 cells) (upper right), healthy late gestation PBMC (n=1, 42 cells) (lower left), preeclampsia decidua delivered on term (n=3, 658 cells) (lower right). (C)-(G) Floating box plots show the abundance of cells in each *FOXP3*$^{+}$ Treg sub- cluster.
